# ELN vs. IPSS-M in MDS/AML: Which Prognostic System Should Guide Clinical Decision-Making?

**DOI:** 10.3390/cancers18182993

**Published:** 2026-09-16

**Authors:** Maria Liga, Angeliki N. Georgopoulou, Dimitrios Tsokanas, Alexandros Spyridonidis, Vassiliki T. Labropoulou

**Affiliations:** 1 Hematology Division and Bone Marrow Transplantation Unit, Institute of Cell Therapy, University of Patras, 26504 Rio, Greece; ligam@upatras.gr (M.L.); angelikigeorgo@gmail.com (A.N.G.); dimitris_tsokanas@yahoo.gr (D.T.); spyridonidis@upatras.gr (A.S.); 2 Hematology Division, Department of Internal Medicine, University Hospital of Patras, University of Patras, 26504 Rio, Greece

**Keywords:** MDS/AML, myelodysplastic syndrome, acute myeloid leukemia, IPSS-M, ELN2022, ELN 2024 Less-Intensive, prognostic scoring systems, molecular classification, risk stratification

## Abstract

The recognition of myelodysplastic syndrome/acute myeloid leukemia (MDS/AML) as a distinct overlap entity by the International Consensus Classification (ICC) has introduced new challenges in prognostication and therapeutic decision-making. Defined by 10–19% blasts in the absence of AML-defining genetic abnormalities, MDS/AML occupies a biological continuum between myelodysplastic syndromes (MDS) and acute myeloid leukemia (AML) and is characterized by unique molecular features, patterns of clonal evolution, and clinical behavior that are not fully captured by prognostic systems originally developed for either disease. Consequently, the emergence of this entity has renewed interest in how existing classification and risk stratification frameworks should be applied to overlap disease. This review examines the biological basis of MDS/AML and critically evaluates the strengths and limitations of the Molecular International Prognostic Scoring System (IPSS-M) and the European LeukemiaNet (ELN) classifications. Available evidence supports IPSS-M as the preferred framework for baseline disease-specific prognostic assessment, with preserved prognostic discrimination in this entity. In contrast, direct application of ELN 2022 results in substantial adverse-risk compression and limited prognostic discrimination. Emerging evidence suggests that disease-specific recalibration and treatment-contextual frameworks, including ELN 2022 modified and ELN 2024 Less-Intensive, may provide additional information, although validation specifically in MDS/AML remains limited and largely retrospective. Collectively, current evidence supports a complementary rather than competitive approach. IPSS-M should provide the disease-specific prognostic foundation, whereas ELN-based assessment may add AML-oriented molecular and treatment-contextual information relevant to therapeutic planning. Neither framework should independently determine treatment intensity. Instead, optimal management should integrate disease-specific prognosis with patient fitness and transplant eligibility, molecular characteristics, treatment context, and dynamic response assessment. Emerging approaches incorporating clonal hierarchy, single-cell analysis, epigenomic and multi-omic profiling, measurable residual disease, and artificial intelligence (AI)- and machine learning (ML)-based predictive models may further refine prognostic assessment, improve prediction of treatment response, and facilitate increasingly individualized therapeutic strategies in MDS/AML, although their clinical application remains investigational.

## Simple Summary

Myelodysplastic syndrome/acute myeloid leukemia (MDS/AML) is a recently recognized overlap entity that exhibits biological and clinical features intermediate between myelodysplastic syndromes and acute myeloid leukemia. Its emergence has challenged the application of traditional prognostic systems developed for either disease. This review examines the biological characteristics of MDS/AML and evaluates the performance of the Molecular International Prognostic Scoring System (IPSS-M) and the European LeukemiaNet (ELN) classifications in this setting. Current evidence supports IPSS-M as the preferred framework for baseline disease-specific prognostic assessment, whereas ELN-based classifications provide complementary AML-oriented molecular and treatment-contextual information. Neither framework should independently determine treatment intensity. Instead, prognosis, patient fitness and transplant eligibility, molecular characteristics, treatment context, and response should be integrated to guide individualized management of patients with MDS/AML.

## 1. Clinical Vignette: The Classification Dilemma

A 72-year-old man presents with transfusion-dependent anemia and thrombocytopenia. Bone marrow examination reveals multilineage dysplasia and 15% myeloblasts. Cytogenetics are normal, while next-generation sequencing identifies ASXL1, RUNX1, and SRSF2 mutations without AML-defining genetic abnormalities. According to the International Consensus Classification (ICC), the patient is diagnosed with MDS/AML, and according to World Health Organization (WHO) 2022 classification, this is categorized as MDS-Increased Blasts-2 (MDS-IB2). However, an immediate clinical dilemma arises: should he be managed as having advanced MDS or AML?

Application of IPSS-M categorizes the patient’s disease as high-risk based on its molecular profile and high blast count, whereas ELN 2022 classifies him as adverse-risk because of myelodysplasia-related mutations. Thus, although both prognostic frameworks evaluate the same underlying disease and molecular profile, they interpret these features within different disease-specific contexts, potentially leading to different risk assignments and therapeutic considerations. An MDS-oriented approach may emphasize hypomethylating therapy, transplantation assessment, and longitudinal molecular monitoring, whereas an AML-oriented strategy may favor venetoclax-based therapy, intensive treatment in selected patients, or enrollment in AML-directed clinical trials.

This case highlights a central question in contemporary myeloid neoplasia: can the choice of classification system influence treatment decisions and, ultimately, patient outcomes? The emergence of MDS/AML suggests that prognosis and therapy may not always be optimally guided by the same framework.

The distinction between myelodysplastic syndromes (MDS) and acute myeloid leukemia (AML) has historically relied on bone marrow blast percentage. Since the adoption of the World Health Organization (WHO) classification, a threshold of ≥20% blasts has served as the principal criterion defining AML, whereas patients below this cutoff have generally been classified as MDS [1,2]. This morphology-based framework provided a practical and reproducible diagnostic system for decades and became deeply embedded in clinical practice, trial design, treatment selection, and prognostic modeling. However, accumulating molecular, genomic, and clinical evidence has increasingly challenged the biological validity of using a fixed blast threshold as the dominant determinant of disease identity [2,3,4,5,6,7,8].

Recognition of these limitations led to the introduction of MDS/AML in the 2022 International Consensus Classification (ICC) of myeloid neoplasms [9]. The ICC formally recognized MDS/AML as a distinct overlap entity characterized by 10–19% blasts in the bone marrow and/or peripheral blood, in the absence of AML-defining recurrent genetic abnormalities [9,10]. According to Hasserjian and colleagues, this category was created to address a long-standing diagnostic gap by recognizing patients who display biological and clinical features intermediate between conventional MDS and AML but are not adequately represented by either diagnostic category alone [10]. Thus, the recognition of MDS/AML represents more than a change in terminology; it reflects a broader movement from morphology-based disease classification toward biologically informed disease definition [10,11,12,13].

Subsequent studies have provided empirical support for the biological validity of this entity. Clinico-genetic, transcriptomic, and comparative classification studies have shown that MDS/AML is not simply MDS with increased blasts or AML below the conventional diagnostic threshold. Rather, it represents a biologically distinct overlap state enriched for myelodysplasia-related biology, characterized by adverse molecular features, clonal evolution patterns resembling advanced MDS, and clinical outcomes that differ from both conventional MDS and overt AML [14,15,16,17,18]. These observations have reinforced the concept that blast percentage alone cannot fully capture disease biology or clinical risk.

The recognition of MDS/AML has created an important prognostic dilemma. Because this entity lies at the interface between MDS and AML, it remains uncertain whether patients should be risk-stratified using prognostic systems developed for MDS, genetic classifications developed for AML, or disease-specific frameworks tailored to overlap disease. This question has direct clinical relevance because prognostic classification influences treatment intensity, transplantation planning, clinical trial eligibility, and interpretation of measurable residual disease.

The Molecular International Prognostic Scoring System (IPSS-M) was developed specifically for MDS and integrates cytopenias, blast percentage, cytogenetics, and recurrent molecular abnormalities into a unified prognostic model [19]. In contrast, the European LeukemiaNet (ELN) classification was developed for AML and stratifies patients according to AML-specific cytogenetic and molecular abnormalities [20]. Although both systems are biologically informed, they were derived from different disease contexts and may not perform equivalently in ICC-defined MDS/AML. The first dedicated comparison by Huber and colleagues demonstrated that IPSS-M provides superior prognostic discrimination compared with ELN 2022 in patients with MDS/AML, raising important questions about how risk should be assessed in this newly recognized entity [11].

This review evaluates the biological and clinical evidence supporting MDS/AML as a distinct overlap entity and critically compares the performance and clinical utility of IPSS-M and ELN-based classifications in this setting. We specifically address whether IPSS-M or ELN should guide prognostic assessment in MDS/AML, how these systems should be integrated into therapeutic decision-making, and why future MDS/AML-specific models may require dynamic incorporation of molecular architecture, clonal evolution, measurable residual disease, treatment context, and multi-omics data.

## 2. MDS/AML: A Distinct Biological Entity at the Interface Between MDS and AML

### 2.1. Biological Continuum Between MDS and AML

The concept that MDS and AML exist along a biological continuum has long been recognized. The French-American-British (FAB) classification included refractory anemia with excess blasts in transformation (RAEB-T), encompassing patients with 20–30% blasts who displayed features intermediate between MDS and AML [21]. However, subsequent WHO classifications eliminated RAEB-T and adopted a uniform threshold of ≥20% blasts for AML diagnosis, thereby dividing what appeared to be a continuous biological process into two distinct diagnostic categories [1,2].

Over the past decade, advances in genomic profiling have increasingly challenged this paradigm. Multiple studies have demonstrated that patients immediately below and above the traditional 20% blast threshold often share similar cytogenetic abnormalities, mutational profiles, clinical characteristics, and survival outcomes [2,3,4,5,6]. These observations suggest that blast percentage alone incompletely reflects disease biology and that myeloid neoplasms evolve through progressive clonal selection and acquisition of genetic abnormalities rather than through abrupt biological transitions occurring at predefined blast percentages [2,3,4,5,6,22,23]. Importantly, population-based sequencing studies have also shown that specific clonal mutations can precede over AML by years and identify individuals at increased future risk of AML [24].

The biological basis for this continuum is rooted in the molecular complexity of MDS. Myelodysplastic syndromes arise from genetically altered hematopoietic stem and progenitor cells that acquire somatic mutations conferring a competitive clonal advantage while disrupting normal hematopoietic differentiation. Disease evolution is characterized by ineffective hematopoiesis, progressive genomic instability, and accumulation of additional genetic lesions that ultimately increase the risk of leukemic transformation [2,3,4,5,6,8]. Large genomic studies have demonstrated that MDS is driven by recurrent mutations affecting RNA splicing, epigenetic regulation, transcriptional control, cohesin function, chromatin remodeling, DNA repair, and signal transduction pathways [4,5,6,25].

Importantly, disease behavior is increasingly recognized as a consequence of complex interactions among co-occurring genetic lesions, clonal architecture, and evolutionary processes rather than the effect of individual mutations considered in isolation. The International Working Group for Prognosis in MDS demonstrated that approximately 94% of patients harbor at least one genetic abnormality and that increasing genomic complexity correlates with disease severity and clinical outcome [19]. These findings provided an important biological rationale for molecularly integrated prognostic systems, while the broader genomic and clinical evidence supporting a continuum between MDS and AML contributed to the recognition of MDS/AML as a distinct overlap entity [9,10,19].

**Figure 1:**
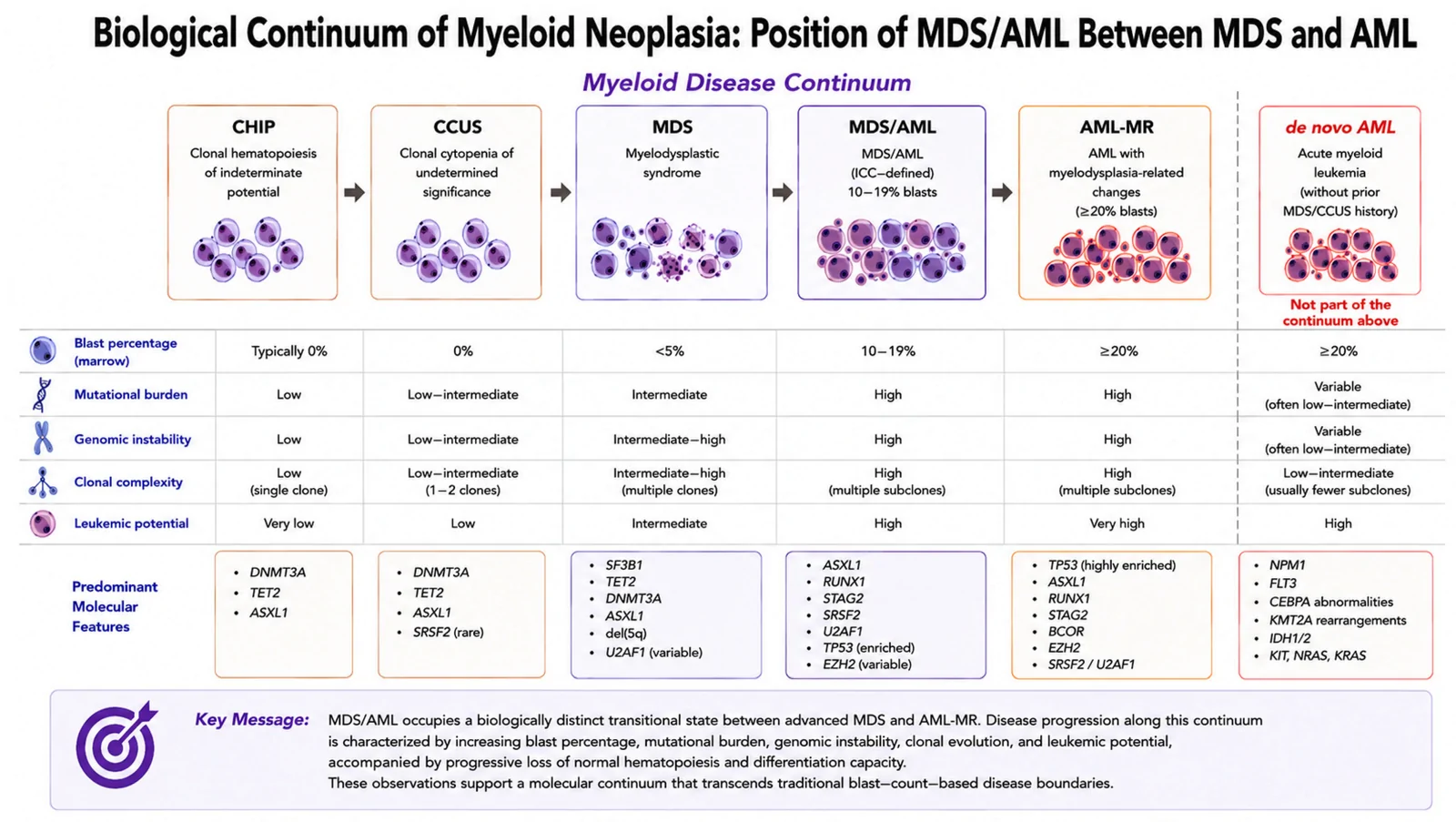
Biological continuum of myeloid neoplasia and the intermediate position of ICC-defined MDS/AML. Disease evolution from CHIP and CCUS through MDS, MDS/AML and AML-MR is characterized by increasing genomic complexity, clonal evolution, and leukemic potential. MDS/AML occupies a distinct intermediate biological state between MDS and AML-MR, supporting biology-based disease classification and prognostication. In contrast, de novo AML lies outside this evolutionary continuum and is characterized by distinct molecular drivers and generally different patterns of genomic complexity compared with AML-MR [3,9,15,17,18,22,23]. Abbreviations: AML, acute myeloid leukemia; AML-MR, acute myeloid leukemia with myelodysplasia-related changes; CCUS, clonal cytopenia of undetermined significance; CHIP, clonal hematopoiesis of indeterminate potential; ICC, International Consensus Classification; MDS, myelodysplastic syndromes.

**Figure 2:**
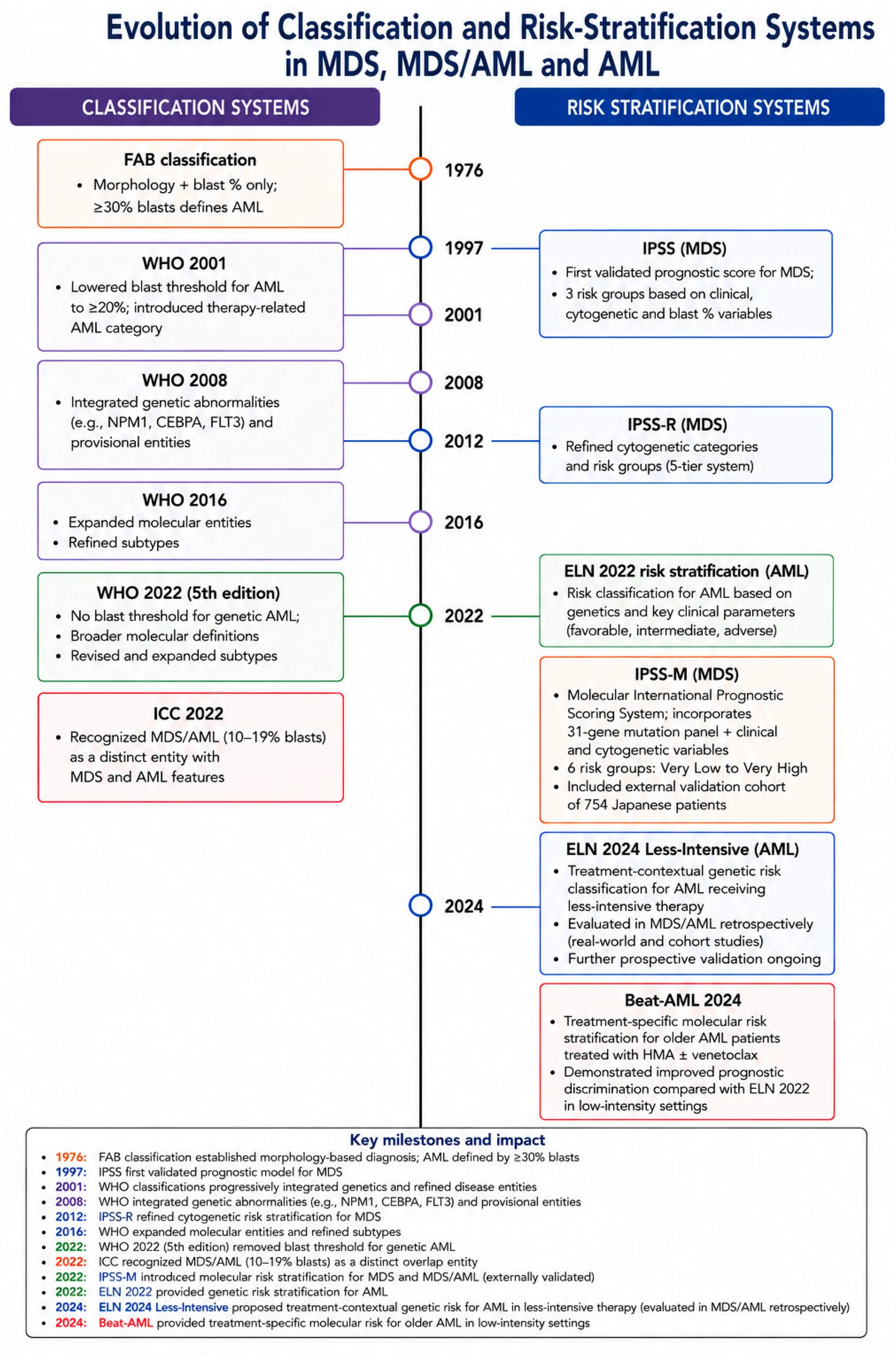
Evolution of classification and prognostic systems in MDS, MDS/AML, and AML. Timeline showing the shift from morphology-based classification (FAB) to molecularly informed classification and risk stratification. WHO 2022 and ICC 2022 recognized MDS/AML as a distinct overlap entity, while IPSS-M, ELN 2024 Less-Intensive, and Beat AML 2024 reflect the move toward disease-specific and treatment-specific prognostic models [1,9,19,20,21,26,27,28,29,30]. Abbreviations: AML, acute myeloid leukemia; ELN, European LeukemiaNet; FAB, French–American–British; HMA, hypomethylating agent; ICC, International Consensus Classification; IPSS, International Prognostic Scoring System; IPSS-M, Molecular International Prognostic Scoring System; IPSS-R, Revised International Prognostic Scoring System; MDS, myelodysplastic syndrome; MDS/AML, myelodysplastic syndrome/acute myeloid leukemia; WHO, World Health Organization.

Accordingly, the ICC introduced MDS/AML as a separate diagnostic category defined by 10–19% blasts in the bone marrow and/or peripheral blood, absence of AML-defining recurrent genetic abnormalities, and evidence of an underlying myelodysplastic neoplasm [9,10]. This definition acknowledges that disease progression represents a gradual process of biological evolution rather than an abrupt transition occurring at a predefined blast threshold. Figure 1 illustrates the biological continuum of myeloid neoplasia and demonstrates the distinct intermediate biological position of ICC-defined MDS/AML between MDS and AML and Figure 2 summarizes the evolution of classification and risk-stratification systems in MDS, MDS/AML, and AML.

### 2.2. Why Is MDS/AML Not Simply Advanced MDS?

The recognition of MDS/AML as a separate category raises an important question: whether patients with 10–19% blasts are biologically and prognostically distinct from advanced MDS with 5–9% blasts. This distinction is particularly relevant because increasing blast burden above 5% is already associated with adverse disease biology and prognosis. Thus, the critical issue is not simply whether MDS/AML differs from MDS overall, but whether the 10% blast threshold identifies a sufficiently distinct biological and clinical state to justify a separate disease designation.

Important evidence addressing this question was provided by Lee and colleagues, who analyzed 716 patients with primary MDS according to the 2022 ICC, including 173 patients reclassified as MDS/AML. Patients with MDS/AML had distinct clinical characteristics and genomic profiles and significantly inferior outcomes compared with patients with blast percentages < 10% [15]. Specifically, MDS/AML was associated with a higher mutational burden and more frequent IDH1, IDH2, ASXL1, BCOR, BCORL1, PHF6, NRAS, RUNX1, GATA2, IKZF1, SRSF2, STAG2, and TP53 mutations, many of which are associated with adverse prognosis. Clinically, patients with MDS/AML had a substantially higher 5-year rate of leukemic transformation (69.3% vs. 23.3%) and markedly shorter median OS (15 vs. 68 months) than the ICC-defined MDS group (both *p* < 0.001) [15]. These findings support the concept that the 10–19% blast group is enriched for adverse molecular and cytogenetic features and has a more aggressive clinical phenotype than MDS with <10% blasts.

However, the comparator group included all MDS cases with <10% blasts, not specifically the 5–9% subgroup. Thus, while the study clearly shows worse outcomes for MDS/AML versus MDS < 10%, it does not prove a sharp biological break at exactly 10% blasts. This is important because adverse molecular features already increase progressively above 5% blasts.

The molecular heterogeneity within MDS/AML itself further argues against defining disease biology by blast percentage alone. Lee et al. demonstrated that MDS/AML with MDS-related gene mutations had outcomes intermediate between MDS/AML with mutated TP53 and MDS/AML, non-otherwise specified (NOS) [15]. Moreover, despite differences in blast percentage, patients with TP53-mutated MDS and MDS/AML showed similar 1-year leukemic transformation rates and nearly identical median OS, emphasizing that dominant molecular abnormalities may transcend blast-defined diagnostic boundaries.

Additional evidence from transcriptomic analyses by Lee and colleagues further demonstrates biological heterogeneity across MDS [16]. Patients without detectable gene mutations or cytogenetic abnormalities had the most favorable outcomes and exhibited immune-metabolic transcriptional programs, whereas genomically altered disease was characterized by inflammatory, proliferative, and stress-response signatures [16]. Increasing genomic complexity was accompanied by progressive transcriptional dysregulation, including activation of TNFα/NF-κB, interferon-response, IL6–JAK–STAT3, KRAS, hypoxia-related, and mitotic spindle pathways. Notably, the 2026 Lee et al. cohort also suggests increasing genomic complexity across adjacent blast-defined categories. Based on the distribution of genomic abnormalities, MDS/AML contained a higher proportion of cases with concomitant gene mutations and cytogenetic abnormalities than WHO-2016 MDS-EB1 (50.8% vs. 38.1%), whereas genomically negative cases were less frequent (3.3% vs. 10.0%). Although these data suggest greater genomic complexity in MDS/AML, the published analysis did not provide a direct gene-by-gene comparison between the 5–9% and 10–19% blast groups.

Additional evidence was provided by Zhang and colleagues, who specifically compared 2028 patients with ICC-defined MDS (<10% blasts) and 374 MDS/AML (10–19% blasts) patients [18]. The two groups showed substantial differences in their mutational landscapes. MDS/AML was enriched for several adverse or myelodysplasia-related alterations, including ASXL1 (31.6%), TP53 (24.9%), STAG2 (22.2%), RUNX1 (21.7%), SRSF2 (20.9%), and BCOR (10.7%). In contrast, lower-blast MDS remained enriched for mutations traditionally associated with more indolent disease biology, including SF3B1 and isolated TET2 alterations [18,31].

These findings suggest that progression from MDS to MDS/AML is associated not merely with quantitative expansion of the malignant clone—increase in blast count—but also with qualitative changes in disease biology, including preferential selection of clones harboring high-risk myelodysplasia-related mutations and increasing molecular complexity [5,18,22,23]. Overall, MDS/AML is clearly associated with higher genetic complexity and worse outcomes than MDS with <10% blasts. However, evidence is insufficient to conclude that the 10% blast threshold represents a discrete biological boundary. Current data instead support a continuum of disease evolution and an important principle of contemporary myeloid neoplasia biology is arising: the prognostic significance of individual mutations is highly context dependent [5,18].

### 2.3. Why Is MDS/AML Not Simply AML?

The converse question is equally important. Despite sharing several adverse molecular features with AML, MDS/AML cannot simply be regarded as AML below the conventional 20% blast threshold.

In a multicenter study evaluating 568 AML and 75 MDS/AML patients, Wei and colleagues demonstrated extensive molecular and clinical convergence between AML with myelodysplasia-related features (AML-MR) and MDS/AML. Both groups were enriched for canonical MR mutations, including ASXL1, TP53, BCOR, and TET2, and exhibited similar cytogenetic profiles characterized by frequent complex karyotypes, chromosome 5q deletions, and chromosome 7 abnormalities [14].

These observations were independently confirmed by Boertjes and colleagues in a large genomic analysis of 2684 intensively treated patients with AML and MDS/AML [17]. By comparing clinically defined secondary AML, molecularly defined AML-MR, and ICC-defined MDS/AML, the investigators identified a highly conserved molecular signature composed of ASXL1, BCOR, ETV6, EZH2, SF3B1, SRSF2, STAG2, U2AF1, and ZRSR2 mutations that characterized both MDS/AML and secondary-type AML [17]. Remarkably, no significant differences in OS were observed between molecularly defined MDS/AML and AML-MR, despite differences in blast count [17]. These findings are consistent with genomic studies identifying distinct molecular subgroups of AML, including a chromatin–spliceosome group characterized by mutations that substantially overlap with those found in myelodysplasia-related disease [32]. Furthermore, patients with MDS/AML and secondary-type AML shared similar clinical characteristics, treatment responses, and long-term outcomes, supporting the concept that these disorders represent biologically related manifestations of a common molecular disease process [14,17].

At the same time, both studies identified important molecular distinctions that justify preservation of MDS/AML as a separate diagnostic category. Wei et al. demonstrated differential enrichment of specific mutations, including BCOR, WT1, and IDH2, between AML-MR and MDS/AML, while Boertjes et al. observed differences in the frequencies of ASXL1, ZRSR2, CBL, and DNMT3A mutations between MDS/AML and secondary-type AML [14,17]. Thus, although the 20% blast threshold did not independently distinguish survival within molecularly defined secondary-type disease, differences in molecular composition persist across the blast-defined groups. Therefore, molecular and prognostic convergence across the 20% blast threshold should not be interpreted as complete biological or clinical equivalence [14,17]. These studies clearly demonstrated that MDS/AML and AML-MR occupy neighboring positions within a common biological spectrum. However, they also identified important differences that support preservation of MDS/AML as a separate disease category.

Particular attention has focused on TP53 abnormalities because their adverse biological and prognostic effects extend across conventional blast-defined disease categories. TP53 alterations, particularly biallelic/multihit abnormalities, are associated with genomic instability, complex cytogenetics, treatment resistance, and poor survival across MDS, MDS/AML, and AML [33,34]. However, outcomes within TP53-mutated disease are not uniform. In patients with TP53-mutated higher-risk MDS and oligoblastic AML treated with eprenetapopt plus azacitidine, substantial response rates were observed, and achievement of response and TP53 molecular clearance was associated with improved overall survival [35]. More broadly, the prognostic impact of TP53 abnormalities is influenced by allelic state, TP53 variant allele frequency (VAF), and cytogenetic complexity, although the relative prognostic contribution of these factors may vary according to disease context [33,34,36,37]. These findings support the concept that TP53-mutated myeloid neoplasms share adverse biological features across conventional MDS and AML boundaries and have prompted efforts to harmonize their classification across contemporary disease frameworks [38]. Both Lee and Wei demonstrated enrichment of TP53-altered disease within MDS/AML [14,15]. Notably, Lee et al. found similarly poor outcomes in TP53-mutated MDS and MDS/AML despite differences in blast percentage, with median OS of 5.2 and 5.1 months, respectively (*p* = 0.905), suggesting that TP53-driven biology may outweigh blast percentage as a prognostic determinant in this molecular subgroup [15]. Nevertheless, TP53-altered myeloid neoplasms remain heterogeneous, with TP53 VAF and cytogenetic risk providing additional prognostic information in patients undergoing allogeneic transplantation [39]. Collectively, available evidence supports substantial biological and prognostic continuity of biallelic/multihit TP53-altered myeloid neoplasms across conventional MDS, MDS/AML, and AML blast thresholds [33,34]. However, whether these disorders should be formally unified into a single molecularly defined entity irrespective of blast percentage remains controversial; current evidence challenges the biological and prognostic primacy of blast-based boundaries but does not yet definitively establish that these diagnostic categories should be abandoned.

Collectively, these findings indicate that MDS/AML cannot simply be regarded as AML below the diagnostic blast threshold. Instead, it occupies an intermediate biological position between advanced MDS and AML-MR, sharing substantial molecular overlap with both diseases while maintaining distinct clinicobiological characteristics [14,15,17,18]. Importantly, Attardi et al. showed that patients with TP53-mutated or myelodysplasia-related cytogenetic disease had similarly poor survival in MDS/AML and AML across the 20% blast threshold. In contrast, among patients with myelodysplasia-related gene-mutated or not otherwise specified (NOS) disease, the 20% blast threshold retained prognostic significance, with MDS/AML showing better survival than AML [40]. The distinct molecular alterations and biological characteristics of ICC-defined MDS/AML, AML-MR, and de novo AML are summarized in Table 1.

The recognition of MDS/AML as a biologically distinct overlap entity has important implications for risk stratification. If MDS/AML is neither simply advanced MDS nor simply AML, it cannot be assumed that prognostic systems developed exclusively within either disease will perform optimally in this setting. These observations provide the biological rationale for evaluating whether MDS-oriented prognostic systems such as IPSS-M or AML-oriented classifications such as ELN more accurately capture prognosis in overlap disease.

This question forms the central focus of the following sections.

## 3. Does Blast Count Still Matter? The Evolution from Morphology to Molecular Prognostication

For decades, bone marrow blast percentage represented a cornerstone of disease classification and prognostic assessment in myeloid neoplasms. The adoption of a 20% blast threshold for AML diagnosis established a practical distinction between MDS and AML and became embedded in clinical practice, clinical trial eligibility, and prognostic modeling [1,2]. Importantly, this threshold also has regulatory implications, because classification as MDS or AML may influence eligibility for therapies approved and labeled specifically for one disease category. Thus, despite increasing recognition of the biological continuum between MDS and AML, the 20% blast threshold retains practical consequences for treatment access and clinical trial enrollment [2,9].

Advances in molecular genetics have challenged the concept that blast percentage alone adequately defines disease biology or prognosis. As discussed in Section 2.2 and Section 2.3, patients across conventional blast-defined boundaries may share similar molecular profiles and outcomes, whereas patients with comparable blast percentages may have markedly different clinical trajectories according to their underlying molecular architecture [3,4,5,14,15,17]. The ICC recognition of MDS/AML with 10–19% blasts reflects this evolving concept and acknowledges a biological continuum between MDS and AML rather than an abrupt transition defined solely by blast percentage [9,10].

Nevertheless, blast count retains important clinical and prognostic value. Increasing blast burden generally accompanies more advanced disease and contributes to established MDS prognostic models [27,28]. Rather than being considered the principal determinant of disease identity, blast percentage may therefore be better viewed as a measure of disease burden and proliferative progression that complements molecular information. Genomic architecture—including high-risk molecular abnormalities, co-mutational patterns, and clonal complexity—provides additional prognostic information and may transcend conventional blast-defined boundaries [5,19,22,23,25].

Accordingly, contemporary classification and prognostication increasingly integrate morphology with molecular genetics rather than replacing one with the other. Blast percentage remains relevant for diagnosis, assessment of disease burden, regulatory treatment eligibility, and clinical trial design, whereas molecular architecture provides increasingly important information regarding disease biology and prognosis. This integrated approach provides the conceptual basis for molecularly informed prognostic systems such as IPSS-M and for the increasing incorporation of genomic features into risk stratification in MDS/AML [11,19].

## 4. Evolution of Prognostic Systems in MDS: From Clinical Variables to Molecular Risk Stratification

The evolution of prognostic systems in MDS mirrors the broader transition from morphology-based disease assessment toward biologically informed risk stratification. The original International Prognostic Scoring System (IPSS), introduced in 1997, represented the first widely adopted standardized framework for predicting survival and risk of leukemic transformation in MDS [27]. The model incorporated three readily available parameters: bone marrow blast percentage, cytogenetic abnormalities, and the number of peripheral blood cytopenias. IPSS significantly improved risk assessment and became the foundation for clinical decision-making, therapeutic stratification, and clinical trial design for more than a decade [27].

In 2012, the Revised International Prognostic Scoring System (IPSS-R) further refined prognostication by expanding cytogenetic risk categories and providing more detailed assessment of blast percentage and cytopenias [28]. Compared with the original IPSS, IPSS-R improved prognostic discrimination and rapidly became the international standard for risk assessment in MDS [28]. Nevertheless, both IPSS and IPSS-R were developed before the routine incorporation of next-generation sequencing and therefore could not account for the prognostic impact of recurrent somatic mutations that are now recognized as major determinants of clinical outcome [28,41,42,43].

During the following decade, genomic studies transformed understanding of MDS biology. Investigations by Papaemmanuil, Bejar, Haferlach, Ogawa, and others demonstrated that mutations affecting RNA splicing, epigenetic regulation, transcriptional control, cohesin function, and signal transduction exert major effects on survival and disease progression independent of conventional prognostic variables [4,41,42,43].

These advances directly led to the development of the Molecular International Prognostic Scoring System (IPSS-M) [19]. Using data from nearly 3000 patients with primary MDS, Bernard and colleagues combined clinical variables, cytogenetics, and mutations in 31 genes to generate the first molecularly integrated prognostic model for MDS [19]. Unlike previous systems, IPSS-M incorporates the biological information contained within recurrent genetic abnormalities and therefore more directly reflects the molecular mechanisms driving disease progression.

A major finding of the original IPSS-M study was the substantial impact of molecular information on risk assessment. Application of IPSS-M resulted in reclassification of approximately 46% of patients compared with IPSS-R, with many patients reassigned to higher- or lower-risk categories based on their molecular profiles [19]. This observation demonstrated that conventional clinical and cytogenetic parameters alone fail to capture a significant proportion of biologically relevant prognostic information.

The development of IPSS-M therefore represents more than a technical refinement of previous scoring systems. Rather, it reflects a fundamental conceptual shift from risk assessment based predominantly on morphology and cytogenetics toward risk stratification driven by disease biology [5,19]. This transition is particularly relevant for MDS/AML, a disease entity characterized by complex molecular architecture and extensive biological heterogeneity. Consequently, the emergence of MDS/AML provides a unique opportunity to evaluate whether a prognostic model specifically developed within myelodysplastic neoplasms can more accurately capture disease behavior than classification systems originally derived from AML populations [11].

## 5. IPSS-M Across MDS and MDS/AML

### 5.1. Independent Validation of IPSS-M in Classical MDS

The prognostic performance of IPSS-M has been independently validated across multiple large and geographically diverse MDS cohorts, including real-world and transplant populations [44,45,46,47]. These studies consistently confirmed its reproducibility and demonstrated improved risk discrimination compared with IPSS-R for overall survival, leukemia-free survival, and leukemic transformation, with molecular information resulting in substantial reclassification of patients across conventional risk categories [44,45,46,47]. Collectively, these validations established IPSS-M as a robust and broadly applicable prognostic framework for MDS and provide the basis for examining the more clinically relevant question addressed in the following section: whether its prognostic performance is maintained in ICC-defined MDS/AML.

### 5.2. Validation of IPSS-M in ICC-Defined MDS/AML

The most important question following the introduction of MDS/AML by the ICC was whether prognostic systems developed for classical MDS would remain applicable in this newly recognized overlap entity [9,10]. Because IPSS-M was derived before formal recognition of MDS/AML, its performance in patients with 10–19% blasts initially remained uncertain. Huber and colleagues provided the first dedicated comparison of MDS- and AML-based prognostic frameworks in ICC-defined MDS/AML, addressing a central clinical question: should MDS/AML be prognostically approached as advanced MDS or as AML [11]?

The investigators analyzed 1451 patients with non–therapy-related myeloid neoplasms, including 137 patients with ICC-defined MDS/AML, all of whom underwent comprehensive genomic characterization [11]. The MDS/AML cohort exhibited a median OS of 2.1 years and was predominantly characterized by MR biology, with 72% harboring MR gene mutations and 14% carrying TP53 mutations and only 10% were classified as not otherwise specified (NOS), underscoring the predominance of myelodysplasia-related biology within this entity [11]. Consistent with previous studies, TP53-mutated disease was associated with particularly poor outcomes, with a median OS of only 0.8 years, whereas the NOS subgroup demonstrated the most favorable prognosis, with a median OS of 8.2 years, further highlighting the major prognostic influence of molecular genetics in overlap disease [11].

Application of IPSS-M distributed patients across multiple risk categories despite the overall high-risk nature of the cohort. Forty-five percent of patients were classified as very high risk, 29% as high risk, and the remainder distributed among intermediate and low-risk groups, whereas no patient was classified as very low risk [11]. Most importantly, IPSS-M achieved highly significant separation of survival outcomes across risk categories (*p* < 0.001), with median OS ranging from 8.9 years in the low-risk group to 1.3 years in the very-high-risk group [11]. Survival within corresponding IPSS-M categories closely resembled that observed in classical MDS cohorts, suggesting that the biological determinants of prognosis in MDS/AML remain largely aligned with those operating in advanced MDS [11].

The prognostic performance of IPSS-M was further supported by concordance analysis. The c-index observed in MDS/AML (0.7125) was virtually identical to that reported in the original MDS reference cohort (0.7155) and a matched MDS population (0.7166), indicating that IPSS-M retains the same prognostic accuracy in overlap disease as in classical MDS [11].

In contrast, application of ELN 2022 produced markedly different results. Ninety-one percent of patients were assigned to the adverse-risk category, only 9% were classified as intermediate risk, and no patient fulfilled criteria for favorable-risk disease [11]. This extreme concentration of patients within a single category substantially reduced prognostic discrimination and reflected the high prevalence of MR mutations that automatically confer adverse-risk status according to ELN 2022 [11,20].

A particularly informative observation was that MDS/AML patients classified as adverse risk according to ELN exhibited substantially longer survival than AML patients assigned to the same risk category. Median OS was 1.9 years in adverse-risk MDS/AML compared with only 0.7 years in adverse-risk AML [11]. These findings indicate that identical molecular abnormalities may not confer the same prognostic implications across different disease entities and suggest that AML-derived risk classifications may overestimate biological aggressiveness in overlap disease.

Beyond validating a prognostic model, the study provided important biological insights. The findings demonstrate that mutational profile and cytogenetic complexity contribute substantially to prognostic heterogeneity in MDS/AML despite the increased blast burden [11]. The superior prognostic discrimination achieved by IPSS-M compared with ELN 2022 further suggests that integration of molecular, cytogenetic, and clinical variables provides more informative risk stratification in MDS/AML than application of an AML-oriented genetic risk classification alone [11].

The successful validation of IPSS-M in ICC-defined MDS/AML has important clinical implications. By preserving prognostic discrimination within this heterogeneous population, IPSS-M enables more refined identification of patients with different expected outcomes and may inform risk-adapted clinical decision-making. Collectively, these findings demonstrate that IPSS-M retains robust prognostic performance in ICC-defined MDS/AML and support its use as an MDS-oriented framework for risk assessment in this overlap population [11].

## 6. ELN Risk Stratification Across AML and MDS/AML

### 6.1. Evolution and Structure of ELN Classifications

The ELN recommendations have become the international standard for genetic risk stratification in AML, providing a unified framework for prognostic assessment, therapeutic decision-making, MRD interpretation, transplantation planning, and clinical trial design in AML [20,48]. Through major revisions in 2017 and 2022, ELN classifications have evolved from predominantly cytogenetic models into increasingly sophisticated molecularly informed systems that integrate recurrent genomic abnormalities with established clinical risk factors [20,48].

The ELN 2022 classification stratifies AML into favorable-, intermediate-, and adverse-risk categories according to recurrent cytogenetic and molecular abnormalities [20]. Favorable-risk AML includes entities such as NPM1-mutated AML without FLT3-ITD, AML with in-frame bZIP CEBPA mutations, and core-binding factor AML. Intermediate-risk AML includes NPM1-mutated AML with FLT3-ITD, wild-type NPM1 AML with FLT3-ITD in the absence of adverse-risk genetic lesions, t(9;11)/MLLT3::KMT2A, and cytogenetic or molecular abnormalities not classified as favorable or adverse. Adverse-risk AML includes complex karyotypes, TP53 mutations, specific chromosomal abnormalities, and AML harboring MR gene mutations involving ASXL1, BCOR, EZH2, RUNX1, SF3B1, SRSF2, STAG2, U2AF1, and ZRSR2 [20]. Importantly, ELN 2022 formally recognized that mutations affecting RNA splicing, chromatin regulation, transcriptional control, and cohesin pathways identify biologically aggressive AML associated with inferior survival and increased relapse risk [20].

Both IPSS-M and ELN provide prognostic information that informs clinical decision-making within their respective disease settings. IPSS-M integrates molecular, cytogenetic, and clinical variables to refine risk stratification in MDS and may inform decisions regarding disease-modifying therapy and transplantation, whereas ELN provides an AML-specific genetic risk framework that contributes to treatment selection, informs response and MRD assessment, and transplantation planning [19,20]. Thus, their principal distinction lies not in whether they guide clinical management, but in the disease populations, prognostic variables, and therapeutic contexts for which they were developed and validated.

### 6.2. Performance and Limitations of ELN in ICC-Defined MDS/AML

Although highly effective in AML, ELN classifications were derived and validated almost exclusively in AML populations, most of whom had ≥20% blasts or AML-defining genetic abnormalities, and therefore assume that the prognostic impact of specific molecular abnormalities remains constant across different disease entities [11,20]. The prognostic significance assigned to individual molecular abnormalities in AML may not be directly transferable to MDS/AML.

As discussed in Section 5.2, Huber et al. demonstrated that application of ELN 2022 to ICC-defined MDS/AML resulted in marked risk-group compression, with the great majority of patients assigned to the adverse-risk category, largely because of the high prevalence of MR gene mutations [11]. Moreover, outcomes within corresponding ELN categories differed between MDS/AML and AML, indicating that ELN 2022 has limited prognostic discrimination when directly applied to MDS/AML [11].

Nevertheless, ELN retains important strengths. Across multiple AML studies, TP53 alterations, complex karyotypes, chromosome 7 abnormalities, and other adverse molecular lesions consistently identify biologically aggressive disease associated with treatment resistance and poor survival [20,33,34]. Thus, although ELN may be less effective as a prognostic instrument in MDS/AML, many of the biological variables it incorporates remain relevant to risk assessment and therapeutic decision-making.

### 6.3. Modified ELN Classifications and Therapeutic Context

The limitations of standard ELN 2022 in MDS/AML prompted the development of a modified ELN classification specifically tailored to this overlap population [49]. The model assigned complex karyotype, −7/del(7q), TP53, RUNX1, and EZH2 alterations to the adverse-risk category, whereas del(5q) and mutations involving ASXL1, SRSF2, U2AF1, ZRSR2, BCOR, and STAG2 were classified as intermediate risk. Favorable risk included isolated SF3B1 mutation or absence of adverse abnormalities [49]. This disease-specific recalibration resulted in reclassification of 37% of patients and a more balanced distribution of favorable-, intermediate-, and adverse-risk disease (16%, 35%, and 49%, respectively), with corresponding median OS of 8.1, 3.1, and 1.3 years (*p* < 0.001) [49].

Treatment context is also increasingly recognized as an important determinant of molecular risk. The ELN 2024 Less-Intensive classification was developed in AML patients receiving less-intensive therapy, particularly venetoclax-based regimens, and differs substantially from ELN 2022 by reducing the adverse prognostic weight of many MR mutations while retaining TP53 as a major adverse determinant [29,50]. Similarly, the Beat-AML 2024 model was developed in older patients with AML receiving lower-intensity therapy and refined ELN 2022 using a mutation score incorporating IDH2, KRAS, MLL2, and TP53 [26]. These models illustrate that the prognostic significance of molecular abnormalities may vary according to treatment context. The original Beat-AML cohort contained 595 patients aged ≥60 years with newly diagnosed AML receiving lower-intensity therapy; ELN 2022 classified 11%, 11%, and 78% as favorable, intermediate, and adverse risk, respectively [26]. Application of the refined Beat-AML 2024 model to 579 evaluable patients redistributed the population into 22% favorable, 41% intermediate, and 37% adverse risk, with corresponding 2-year OS rates of 48%, 33%, and 11%, respectively [26].

More recently, Hu et al. evaluated IPSS-M, ELN 2022, and ELN 2024 in 110 patients with ICC-defined MDS/AML. ELN 2022 classified 79.1% of the overall cohort as adverse risk. Among 62 patients receiving HMA-based therapy, ELN 2024 classified 42/62 (67.7%) as favorable, 7/62 (11.3%) as intermediate, and 13/62 (21.0%) as adverse risk, with median OS of 33, 18, and 12 months, respectively (*p* = 0.005) [51]. ELN 2024 showed numerically greater prognostic discrimination than IPSS-M in this treatment-defined subgroup, although the difference was not statistically significant [51].

For comparison, Kewan et al. evaluated 426 patients with AML and reported favorable-, intermediate-, and adverse-risk distributions of 20%, 20%, and 60% with ELN 2022; 55%, 24%, and 20% with ELN 2024; and 21%, 38%, and 41% with Beat-AML 2024, respectively [52]. The relative performance of these models also varied according to treatment intensity, with ELN 2024 and Beat-AML 2024 better identifying favorable-risk patients receiving less-intensive therapy [52]. In contrast, Beat-AML 2024 has not yet been specifically validated in a dedicated ICC-defined MDS/AML cohort, precluding a direct comparison of its risk-group distribution between MDS/AML and AML.

Collectively, these observations reinforce a central principle of contemporary molecular hematology: the prognostic significance of molecular abnormalities is both disease- and treatment-context dependent. IPSS-M remains a well-validated disease-specific prognostic framework for MDS/AML, while disease-specific modification of ELN also improves risk discrimination [11,49]. Emerging evidence suggests that ELN 2024 Less-Intensive may provide additional treatment-contextual prognostic information in HMA-treated MDS/AML [51], whereas the applicability of Beat-AML 2024 to MDS/AML remains to be established.

Table 2 summarizes the principal features of IPSS-M, ELN 2022, the modified ELN classification for MDS/AML, and the ELN 2024 Less-Intensive classification, while Figure 2 illustrates the chronological evolution of classification and risk-stratification systems from morphology-based frameworks to contemporary molecularly and treatment-contextual models in MDS, MDS/AML, and AML.

## 7. Which Prognostic Framework Should Be Used in MDS/AML?

**Figure 3:**
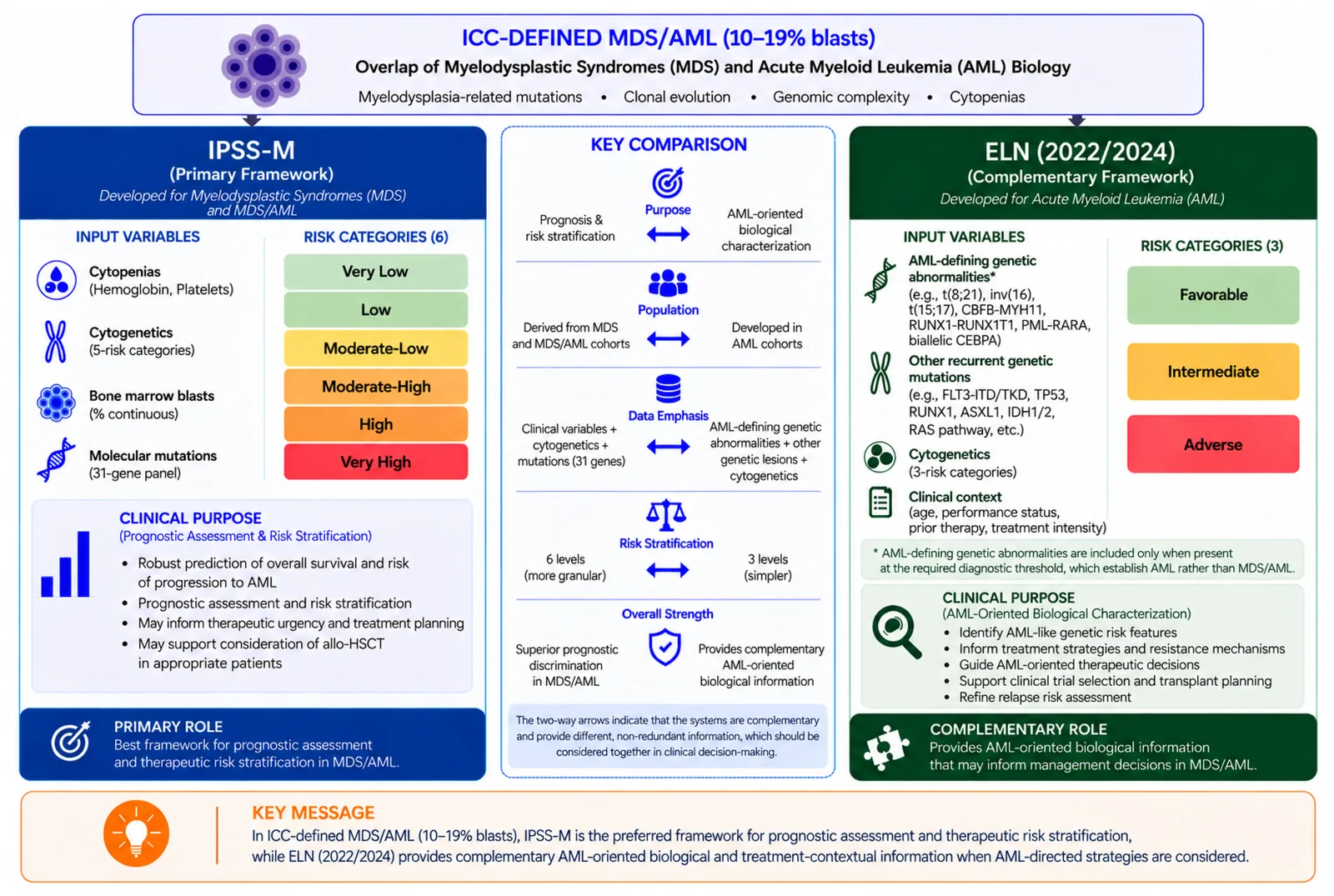
Complementary roles of IPSS-M and ELN in MDS/AML. IPSS-M serves as the primary framework for prognostic assessment and risk stratification in ICC-defined MDS/AML, whereas ELN-based classifications provide complementary AML-oriented biological and treatment-contextual information that may inform therapeutic planning, MRD interpretation, clinical trial eligibility, and transplantation strategy. Together, these frameworks support integrated clinical decision-making, although neither should independently determine treatment intensity in MDS/AML [9,11,19,20,29,51]. Abbreviations: ELN, European LeukemiaNet; ICC, International Consensus Classification; IPSS-M, Molecular International Prognostic Scoring System; MDS/AML, myelodysplastic syndrome/acute myeloid leukemia overlap.

Current evidence supports IPSS-M as the preferred framework for baseline disease-specific prognostic assessment in ICC-defined MDS/AML, because it preserves prognostic discrimination across this heterogeneous overlap population and incorporates clinical, cytogenetic, blast, and molecular variables relevant to disease outcome [11,19,51]. In contrast, ELN-based classifications should be regarded as complementary rather than competing frameworks: ELN 2022 provides an AML-oriented molecular context, although its direct application to MDS/AML is limited by substantial adverse-risk compression, whereas ELN 2024 Less-Intensive may provide additional treatment-contextual prognostic information when less-intensive AML-type therapies are considered, although its validation in MDS/AML remains preliminary and retrospective [11,20,29,51]. Thus, neither IPSS-M nor ELN should independently determinetreatment intensity; rather, IPSS-Mshould provide the disease-specific prognostic foundation, with ELN-based assessment adding AML-oriented molecular and treatment-contextual information that may inform therapeutic planning, transplantation considerations, response assessment, and eligibility for selected clinical trials [11,19,20,29]. Figure 3 illustrates these complementary roles, while the following section translates this concept into a practical clinical framework in which disease-specific prognosis, patient fitness and transplant candidacy, molecular/ELN context, treatment selection, and dynamic response assessment are sequentially integrated.

## 8. How Should MDS/AML Patients Actually Be Treated?

### 8.1. MDS/AML at the Therapeutic Interface Between MDS and AML

Historically, MDS and AML have been managed according to distinct therapeutic paradigms. MDS treatment has largely relied on risk-adapted approaches including supportive care, erythropoiesis-stimulating agents, immunomodulatory therapies, hypomethylating agents (HMAs), and allogeneic hematopoietic stem-cell transplantation (allo-HSCT) for higher-risk disease, whereas AML management has focused on intensive chemotherapy, molecularly targeted therapies, transplantation-based consolidation, while venetoclax-based lower-intensity regimens have more recently assumed an increasingly important role, particularly in patients considered unsuitable for intensive chemotherapy [53,54,55,56,57].

The recognition of MDS/AML as a distinct overlap entity challenges this traditional separation. Most MDS/AML cases retain substantial myelodysplasia-related molecular biology, yet their increased blast burden and risk of leukemic progression may justify consideration of AML-oriented strategies in selected patients [14,15,18]. Importantly, however, treatment should not be determined by blast percentage or diagnostic terminology alone. Therapeutic selection should integrate disease-specific prognosis, molecular and cytogenetic features, physiologic fitness, transplant eligibility, actionable molecular abnormalities, prior therapy, regulatory considerations, treatment intent, and patient preferences.

### 8.2. Integrating IPSS-M and ELN into Therapeutic Decision-Making

The complementary use of IPSS-M and ELN-based classifications in MDS/AML should not be interpreted as assigning different prognostic systems according to treatment intensity. Current evidence supports IPSS-M as the principal disease-specific prognostic framework in ICC-defined MDS/AML because it retains prognostic discrimination across the heterogeneous population with 10–19% blasts [11,19]. In clinical practice, IPSS-M therefore addresses the fundamental question: “What is this patient’s baseline disease-related risk?” Higher IPSS-M categories identify patients with an increased risk of disease progression and death and can support decisions regarding the urgency of disease-modifying therapy and early evaluation for allogeneic hematopoietic stem-cell transplantation [19,58,59]. Importantly, however, IPSS-M is a prognostic rather than treatment-predictive model and should not, by itself, determine whether an individual patient receives intensive AML-type therapy or a lower-intensity regimen.

ELN-based assessment addresses a related but different question: “Does the molecular profile provide additional AML-oriented or treatment-contextual information relevant to management?” In MDS/AML, abnormalities such as TP53, FLT3, IDH1/2, RAS-pathway mutations, and myelodysplasia-related gene mutations may provide information regarding disease biology, potential therapeutic targets, anticipated treatment response or resistance, transplantation strategy, and eligibility for selected clinical trials [20,29,34,50]. However, these abnormalities should not be interpreted as evidence that the patient would derive greater benefit from intensive AML-type chemotherapy. In particular, although KRAS, NRAS, and FLT3 mutations may provide important prognostic or treatment-contextual information, they have not been prospectively demonstrated to predict preferential benefit from intensive induction therapy over lower-intensity approaches in patients with ICC-defined MDS/AML.

The development of the ELN 2024 Less-Intensive classification and treatment-specific models such as Beat-AML further illustrates the importance of treatment context [26,29]. In AML populations receiving less-intensive therapy including venetoclax-based regimens, outcomes vary substantially according to molecular profile. TP53 and signaling-pathway abnormalities, including FLT3 and RAS-pathway mutations, have been associated with inferior outcomes, whereas selected IDH1/2- and DDX41-mutated subsets show more favorable outcomes [29]. These findings demonstrate that the prognostic significance of individual molecular lesions may depend on the therapeutic context; however, they were derived predominantly from AML cohorts and should not be extrapolated as evidence for selecting intensive AML-type chemotherapy in MDS/AML. Whether specific molecular subsets of MDS/AML derive preferential benefit from intensive AML-directed therapy versus lower-intensity approaches remains an important question that requires prospective evaluation.

Accordingly, IPSS-M and ELN-based approaches should be regarded as complementary rather than competing frameworks. IPSS-M provides the primary disease-specific estimate of prognosis in MDS/AML, whereas AML-oriented molecular assessment may add treatment-contextual information concerning actionable targets, expected sensitivity or resistance to particular therapies, transplantation considerations, and clinical-trial options. Neither framework should independently dictate treatment intensity. Therapeutic decisions should instead integrate disease-specific risk, molecular profile, physiologic fitness, transplant eligibility, prior therapy, therapeutic intent, regulatory considerations, and patient preference [11,19,20,29].

### 8.3. A Practical Framework for Treatment Selection and Transplantation

Translation of molecular risk into treatment requires sequential integration of disease- and patient-related factors. A practical approach can be organized into five steps: (1) disease-specific prognostication with IPSS-M; (2) assessment of physiologic fitness and transplant candidacy; (3) interpretation of the molecular profile within an AML-oriented context when appropriate; (4) individualized treatment selection; and (5) dynamic reassessment according to treatment response and MRD when informative. IPSS-M should serve as the principal disease-specific prognostic framework in ICC-defined MDS/AML, whereas treatment feasibility and transplant candidacy should be assessed independently according to performance status, comorbidity burden including HCT-CI, organ function, frailty and functional reserve, donor availability, and patient preferences rather than chronological age alone [11,19,58,60]. For higher-risk, transplant-eligible patients, early donor identification and a strategy aimed at achieving adequate disease control before allo-HSCT should therefore be considered.

**Figure 4:**
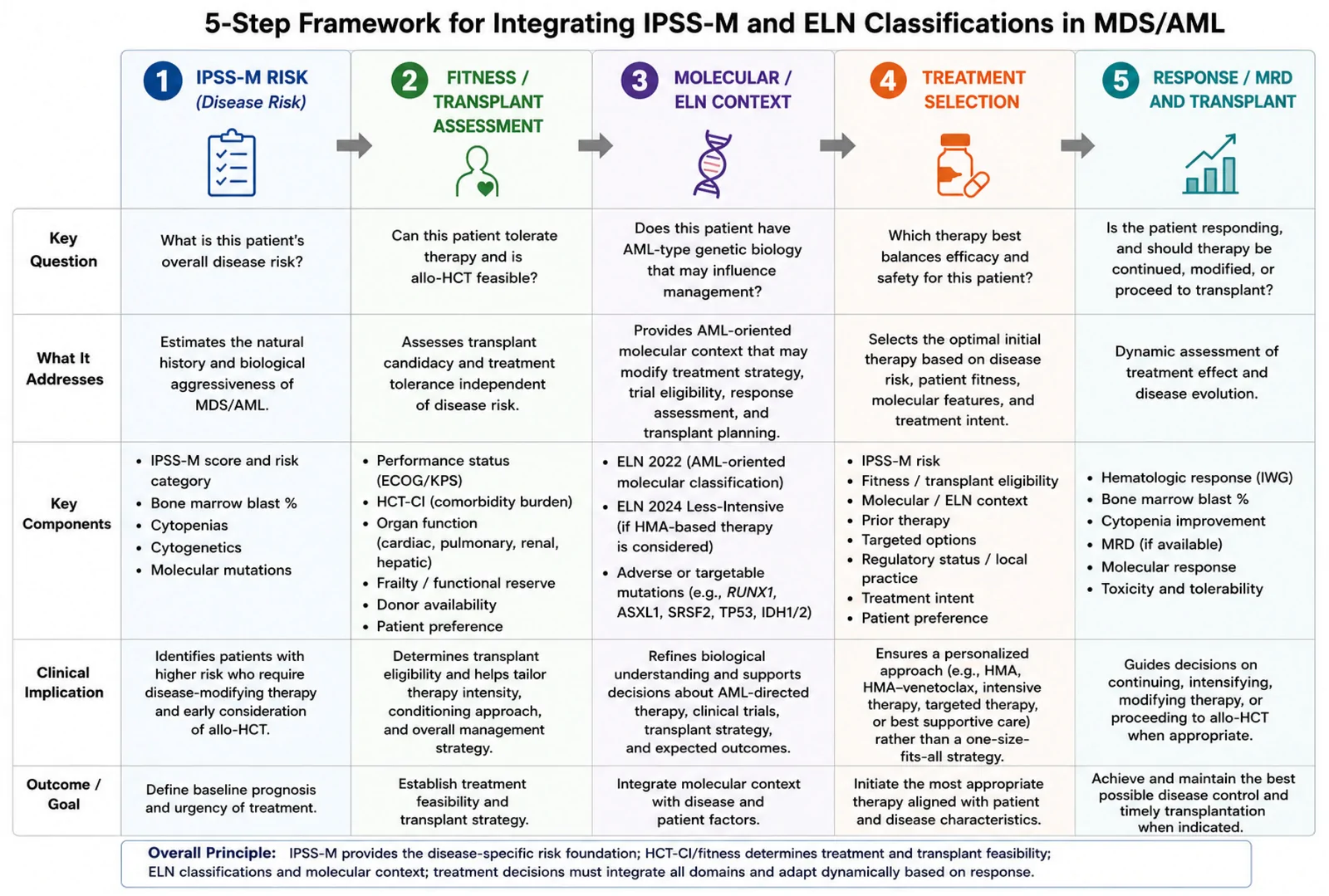
Integrated five-step framework for clinical decision-making in MDS/AML. The proposed approach combines disease-specific prognostication with patient fitness and transplant assessment, AML-oriented molecular context, individualized treatment selection, and dynamic response evaluation. IPSS-M serves as the principal prognostic framework, whereas ELN-based assessment provides complementary molecular and treatment-context information when AML-directed strategies are considered. Treatment decisions should integrate disease biology, patient characteristics, therapeutic intent, and response/MRD, with consideration of allo-HCT when appropriate [11,12,19,20,29,54,57,58,60,61,62]. Abbreviations: ELN, European LeukemiaNet; HCT-CI, Hematopoietic Cell Transplantation specific Comorbidity Index; HMA, hypomethylating agent; ICC, International Consensus Classification; IPSS-M, Molecular International Prognostic Scoring System; MDS/AML, myelodysplastic syndrome/acute myeloid leukemia overlap; IWG, International Working Group.

ELN-based classifications may subsequently provide complementary treatment-contextual information but should not replace IPSS-M for MDS/AML prognostication. The molecular profile should be interpreted in an AML-oriented context when relevant. ELN 2022 may be informative when AML-directed intensive therapy is considered by identifying genetic features associated with adverse AML biology, whereas ELN 2024 Less-Intensive may provide additional treatment-specific prognostic context when less-intensive AML regimens, particularly HMA–venetoclax-based therapy, are used [20,29]. Recent retrospective data have provided preliminary evidence of ELN 2024 Less-Intensive applicability to ICC-defined MDS/AML [51]. Accordingly, ELN 2024 may provide complementary treatment-contextual prognostic information in HMA-treated MDS/AML, but prospective validation is required before it can be used to guide treatment selection [51]. Neither ELN framework should currently be used independently to determine treatment intensity. Treatment selection should ultimately integrate IPSS-M risk, physiologic fitness, transplant eligibility, molecularly actionable abnormalities, prior therapy, regulatory considerations, therapeutic intent, and patient preferences, followed by dynamic reassessment according to treatment response and MRD when clinically informative [11,19,20,29]. Thus, a practical sequence is: diagnosis → IPSS-M risk → fitness/transplant assessment → molecular/ELN context → treatment selection → response/MRD → allo-HSCT when appropriate. Figure 4 illustrates a proposed framework for integrating IPSS-M and ELN-based classifications into risk stratification and clinical management of ICC-defined MDS/AML.

### 8.4. Clinical Application: Revisiting the MDS/AML Vignette

The clinical vignette presented at the beginning of this review illustrates this approach. “A 72-year-old man presents with transfusion-dependent anemia and thrombocytopenia. Bone marrow examination reveals multilineage dysplasia and 15% myeloblasts. Cytogenetics are normal, while next-generation sequencing identifies ASXL1, RUNX1, and SRSF2 mutations without AML-defining genetic abnormalities”. According to the ICC, the patient is diagnosed with MDS/AML.

Step 1—Disease-specific risk assessment with IPSS-M: The presence of 15% marrow blasts together with ASXL1, RUNX1, and SRSF2 mutations suggests adverse disease biology; however, assignment to a specific IPSS-M category requires complete hematologic, cytogenetic, and molecular data. Once calculated, IPSS-M provides the baseline estimate of disease-related prognosis and helps inform the urgency of disease-modifying therapy and early consideration of allo-HSCT [11,19].

Step 2—Fitness and transplant candidacy: Because disease risk alone does not determine treatment feasibility, physiologic fitness and transplant candidacy should be assessed independently. This assessment should incorporate performance status, HCT-CI, organ function, frailty and functional reserve, donor availability, and patient preferences rather than chronological age alone [60]. Importantly, fitness for intensive therapy and eligibility for allo-HSCT should be considered separately, as a patient who is not an appropriate candidate for intensive induction may still be eligible for transplantation following an effective lower-intensity treatment approach.

Step 3—AML-oriented molecular context: Under ELN 2022, ASXL1, RUNX1, and SRSF2 are MR gene mutations associated with adverse-risk AML biology [20]. Their presence in this patient identifies a secondary/MR-type molecular signature and provides complementary biological and treatment-contextual information relevant to therapeutic planning, clinical-trial eligibility, response assessment, and transplantation strategy [14,17,20]. However, the presence of these MR mutations should not by itself be interpreted as an indication for intensive AML-type therapy, as their predictive value for selecting treatment intensity in ICC-defined MDS/AML has not been established.

Step 4—Treatment selection: Treatment selection should integrate IPSS-M risk, physiologic fitness, transplant eligibility, actionable molecular abnormalities, prior therapy, regulatory considerations, therapeutic intent, and patient preferences [54,56]. If the patient is unsuitable for intensive induction, an HMA-based strategy represents a reasonable therapeutic backbone; HMA-based combinations, including venetoclax-containing approaches, may be considered in selected patients according to available evidence, regulatory status, local practice, and preferably within a clinical trial when not established specifically for MDS/AML [54,57]. In this setting, ELN 2024 Less-Intensive may provide additional treatment-contextual prognostic information, although its application in MDS/AML is currently supported by retrospective evidence and should not independently determine treatment selection [51]. If the patient is physiologically fit and transplant eligible, treatment should instead be planned within a transplant-oriented strategy, with the objective of achieving adequate disease control followed by allo-HSCT when appropriate [58].

Step 5—Dynamic reassessment according to treatment response: Baseline prognostic classification should not be regarded as static. Treatment response, evolution of cytopenias and marrow blast burden, acquisition or clearance of molecular abnormalities, and MRD when clinically informative should be incorporated into subsequent therapeutic decisions [61,62]. In a transplant-eligible patient, adequate disease control should trigger reassessment for allo-HCT rather than simply continuation of the initial treatment strategy [58,62].

### 8.5. Lessons from VERONA: Prognostic Risk Does Not Necessarily Predict Treatment Benefit

The phase III VERONA trial has important implications for therapeutic stratification at the MDS/AML therapeutic interface. Azacitidine has long served as a benchmark therapy for higher-risk MDS, with systematic trial reviews documenting the historical response and survival framework against which newer combinations have been evaluated [63]. Although an earlier phase Ib study of azacitidine plus venetoclax in treatment-naïve higher-risk MDS reported a modified overall response rate of 80.4% and median OS of 26.0 months, VERONA failed to demonstrate an OS benefit for venetoclax plus azacitidine over azacitidine alone [57,64]. Differences in study populations may have contributed to these discordant results: the phase Ib study was enriched for high/very-high-risk and excess-blast disease, whereas VERONA included a broader higher-risk MDS population [57,64]. Whether blast burden or specific molecular profiles identify patients more likely to benefit from venetoclax therefore remains unresolved.

Recent real-world data support this biological heterogeneity. In the International Consortium for MDS VALIDATE analysis of 1907 patients with higher-risk MDS, HMA–venetoclax improved composite complete remission compared with HMA alone (48.8% vs. 27.7%; *p* < 0.001) but did not significantly improve OS in the overall population (HR 0.83, 95% CI 0.64–1.07; *p* = 0.15) [65]. However, an OS benefit was observed in TP53-wild-type disease (HR 0.47, 95% CI 0.29–0.74; *p* = 0.002), while patients with ≥10% marrow blasts showed a trend toward improved OS (HR 0.73, 95% CI 0.53–1.01; *p* = 0.06) [65]. The latter observation is particularly relevant to the 10–19% blast range of ICC-defined MDS/AML, but remains hypothesis-generating because MDS/AML was not evaluated as a distinct subgroup.

These findings reinforce an important distinction between prognostic and treatment-predictive stratification. IPSS-M provides disease-specific prognostic information and retains discrimination in MDS/AML [11,19,51], whereas emerging evidence suggests that ELN 2024 Less-Intensive may provide additional treatment-contextual prognostic stratification in HMA-treated MDS/AML [51]. However, neither model has been validated to predict benefit from venetoclax specifically in ICC-defined MDS/AML. Thus, neither a high IPSS-M category nor an adverse ELN designation should independently determine the use of venetoclax. Collectively, VERONA and subsequent data highlight the need for treatment-specific predictive models integrating molecular profile, TP53 status, blast burden, and therapeutic context in MDS/AML [57,64,65].

### 8.6. Clinical Trial Eligibility in MDS/AML: Bridging the MDS–AML Therapeutic Divide

Clinical-trial eligibility represents another potential interface between MDS/AML and AML-directed management. Although many AML trials retain conventional AML eligibility criteria and may exclude patients with 10–19% blasts, several contemporary studies explicitly permit ICC-defined MDS/AML. Current examples include trials evaluating CER-1236 (NCT06834282), HC-7366 (NCT06285890), cirtuvivint-based therapy (NCT06484062), TCB008 (NCT05358808), Q702 plus azacitidine–venetoclax (NCT06445907), and regorafenib plus venetoclax–azacitidine (NCT06454409), all of which allow patients with MDS/AML or 10–19% blasts in specified cohorts. Thus, recognition of MDS/AML may expand access to selected AML-oriented investigational strategies; however, eligibility remains protocol-specific and should not be inferred from ELN risk assignment alone [66,67,68,69,70,71].

## 9. Future Directions: Toward MDS/AML-Specific Prognostic and Therapeutic Decision Models

The recognition of MDS/AML as a distinct overlap entity highlights the limitations of prognostic systems based predominantly on baseline variables. Although IPSS-M currently provides robust disease-specific prognostication, MDS/AML is biologically dynamic, with ongoing clonal evolution and treatment-driven selection [11,19,22,23]. Future models should therefore move beyond static baseline risk toward integration of clonal architecture, multi-omics, MRD, treatment response, and longitudinal clinical information.

### 9.1. Mutation Hierarchy, Clonal Evolution, and Single-Cell Approaches

How does the disease evolve?

One of the most important lessons from contemporary genomic studies is that prognosis cannot be adequately explained by individual mutations alone. Disease behavior is influenced by the broader molecular context in which genetic lesions occur, including co-mutational interactions, clonal hierarchy, mutation acquisition order, allelic burden, and evolutionary dynamics [5,23,72]. Importantly, mutation acquisition is non-random, and the biological consequences of a given alteration may differ according to whether it represents an early founder event or a later subclonal lesion. Nagata et al. demonstrated reproducible patterns of clonal succession in MDS, supporting the contribution of mutation hierarchy and acquisition order to disease phenotype and clinical outcome [72].

This concept is especially relevant in MDS/AML, which typically develops through progressive clonal selection originating from antecedent myelodysplasia [15,23]. As a result, patients frequently harbor multiple cooperating molecular lesions whose biological effects depend on the surrounding genomic landscape. Studies by Bernard and colleagues demonstrated that clinically meaningful disease subgroups emerge from combinations of genomic abnormalities rather than isolated mutations [5]. Although bulk sequencing can estimate variant allele frequencies and infer aspects of clonal architecture, it cannot reliably determine which mutations coexist within individual cells or fully reconstruct complex evolutionary trajectories. Single-cell approaches overcome this limitation by directly resolving clonal architecture and evolutionary trajectories. Chen et al. demonstrated nonlinear and parallel clonal evolution at the stem-cell level during progression from MDS to AML, while Guess et al. and Miles et al. directly identified distinct patterns of clonal evolution and mutational co-occurrence in myeloid malignancies [73,74,75]. More recently, single-cell multi-omics integrating genotype and immunophenotype has shown the ability to distinguish clonal hematopoiesis/preleukemic clones from leukemia-associated clones and to identify patient-specific molecular targets in AML [76].

These approaches may ultimately refine the biological characterization of MDS/AML and clarify its relationship with AML-MR and other AML subtypes. However, no validated single-cell or multi-omic classifier currently distinguishes ICC-defined MDS/AML from AML-MR or other AML categories for routine diagnosis. Their current role therefore remains primarily investigational, providing insight into clonal architecture, disease evolution, and potentially individualized molecular monitoring rather than replacing established diagnostic criteria [73,74,75,76]. The recent study supporting this concept involved only six AML patients, underscoring the need for validation in larger disease-specific cohorts [76].

Future prognostic models will therefore need to evaluate disease biology as an integrated molecular ecosystem rather than as a collection of independent mutations, particularly in MDS/AML where clonal evolution is a defining biological feature.

### 9.2. Multi-Omics and Epigenomic Profiling

Can we characterize its biology more completely?

Although genomic profiling has transformed risk assessment, genomic information alone captures only part of the biological processes driving disease progression and therapeutic response. Patients with similar mutational profiles may experience markedly different clinical outcomes, suggesting that additional biological layers contribute to disease behavior [5,16,77].

Transcriptomic studies have provided important evidence supporting this concept. Lee and colleagues demonstrated that gene-expression profiling improves risk stratification across IPSS-R, IPSS-M, and ICC-defined disease categories and remains independently associated with both OS and LFS after adjustment for established risk factors [16]. These findings indicate that transcriptional programs capture biological information beyond recurrent mutations and cytogenetic abnormalities.

Epigenomic profiling represents another emerging approach for refining disease classification. Single-cell DNA methylation analysis has demonstrated dynamic epigenetic changes during transformation from MDS to AML and identified epigenetic pathways associated with leukemic progression [78]. Complementarily, Steinicke et al. demonstrated that DNA methylation signatures can rapidly distinguish acute leukemia subtypes and resolve diagnostically challenging cases, illustrating the ability of epigenomic patterns to capture lineage and differentiation states beyond conventional genomic classification [79]. Although these approaches have not yet been specifically validated for risk stratification in ICC-defined MDS/AML, they provide a strong rationale for evaluating methylation-based classification in this overlap entity [78,79].

Future predictive models will likely integrate multiple layers of biological information, including genomic, transcriptomic, epigenomic, proteomic, single-cell, and functional biomarkers. Such multi-omics approaches may improve biological classification and prediction of disease progression and therapeutic response beyond currently available baseline risk models [16,78,79,80].

### 9.3. Measurable Residual Disease and Dynamic Risk Assessment

How does risk change during treatment?

A major limitation of current prognostic systems is that they provide a static estimate of risk at diagnosis and therefore may not fully capture the biological changes that occur during disease evolution or treatment. This limitation is particularly relevant in MDS/AML, where clonal architecture continuously changes over time [22,23,72].

In AML, MRD has emerged as one of the strongest predictors of relapse, treatment response, and survival, often surpassing baseline genetic risk classification [20,61]. However, MRD assessment in MDS/AML requires particular consideration because many well-established molecular MRD markers in AML correspond to AML-defining genetic abnormalities and therefore have limited applicability to ICC-defined MDS/AML [9,61].

Multiparameter flow cytometry (MFC) provides a broadly applicable approach independent of a specific molecular marker. Orvain et al. evaluated pre-transplant MFC-MRD in 151 patients with MDS/AML undergoing allo-HCT and demonstrated prognostic associations with post-transplant relapse, RFS, and OS, although its prognostic impact was weaker than in AML [62]. Molecular monitoring by NGS may also be informative when a suitable patient-specific lesion is available, but interpretation depends strongly on the mutation involved. Persistent DNMT3A, TET2, or ASXL1 (DTA) mutations frequently represent antecedent clonal hematopoiesis and should not, in isolation, be considered evidence of residual disease. In contrast, persistence or re-emergence of signaling mutations such as FLT3, NRAS, KRAS, or KIT is more suggestive of residual malignant disease; however, these mutations are often subclonal and may be lost during disease evolution, limiting their negative predictive value. Other non-DTA mutations may also be informative when clearly linked to the disease clone, particularly when serial monitoring demonstrates persistence or increasing variant allele frequency, but their significance remains mutation- and context-dependent [61].

Accordingly, MFC and patient-specific molecular monitoring may provide complementary approaches to dynamic disease assessment, particularly in the peri-transplant setting. The IWG 2023 response criteria nevertheless regard MRD as a provisional endpoint in higher-risk MDS because methodology and clinical utility remain insufficiently standardized [81]. Prospective studies are therefore required before MRD can routinely guide therapeutic decisions specifically in ICC-defined MDS/AML [62,81].

Future MDS/AML-specific models will likely incorporate serial molecular monitoring and MRD assessment as continuously updated variables. Such approaches could facilitate earlier identification of treatment failure and molecular relapse while supporting more individualized therapeutic decision-making throughout the disease course [61,62,81].

### 9.4. Artificial Intelligence and Machine Learning

How can these complex data be integrated?

Artificial intelligence (AI) and machine-learning (ML) methodologies represent one of the most promising developments in the future of risk stratification for myeloid malignancies. Unlike conventional prognostic systems, ML approaches can integrate large numbers of clinical, cytogenetic, genomic, transcriptomic, and treatment-related variables while identifying complex nonlinear relationships [80,82].

Awada and colleagues demonstrated that ML algorithms can identify biologically meaningful genomic subclasses extending beyond traditional disease classifications, with distinct molecular characteristics and clinical outcomes [82]. More recently, Clichet and colleagues demonstrated that an AI-based flow-cytometry MDS score closely correlates with contemporary 2022 disease classifications and IPSS-M, supporting the potential complementary role of AI-based approaches in MDS risk assessment [83].

As highlighted by Al-Nusair and colleagues, AI is increasingly being applied to multi-omics datasets, digital pathology, flow cytometry, MRD assessment, and longitudinal clinical data [80]. In MDS/AML, these approaches may facilitate identification of previously unrecognized biological subgroups, prediction of disease evolution, and generation of individualized risk assessments that adapt over time [80,82,83].

### 9.5. Toward Dedicated MDS/AML-Specific Predictive Frameworks

Can we predict which treatment will benefit an individual patient?

The successful validation of IPSS-M in ICC-defined MDS/AML represents an important advance, but prognostic discrimination does not necessarily establish treatment prediction [11,19]. Future MDS/AML-specific models should therefore move beyond estimating survival toward predicting the probability of benefit from specific therapeutic strategies.

Such models could integrate baseline molecular risk with clonal architecture, multi-omic signatures, treatment-specific biomarkers, MRD kinetics, treatment response, transplantation-related variables, and patient characteristics [16,62,72,73,74,75,76,78,79,81]. Their ultimate objective should be to distinguish not only which patients have poor prognosis, but which patients are most likely to benefit from a particular intervention, including HMA-based combinations, targeted therapies, allo-HCT, or molecularly selected clinical trials [57,58,65].

The development of these disease-specific frameworks represents a major priority for future research and may ultimately establish MDS/AML as a paradigm for precision hematology in the genomic era [16,18,80,83].

## 10. Conclusions

The recognition of MDS/AML as a distinct overlap entity by the ICC reflects the transition from blast-based classification toward biologically informed assessment of myeloid neoplasms. MDS/AML is characterized by substantial molecular heterogeneity and enrichment for myelodysplasia-related abnormalities, while sharing biological features with both advanced MDS and AML-MR. These observations support its position within a disease continuum in which molecular architecture, rather than blast percentage alone, is a major determinant of outcome.

Current evidence supports IPSS-M as the preferred framework for disease-specific prognostication in ICC-defined MDS/AML, whereas ELN-based classifications provide complementary AML-oriented molecular and treatment-contextual information. Importantly, ELN should not replace IPSS-M or independently determine treatment intensity. Clinical decisions should integrate disease risk with physiologic fitness, transplant candidacy, actionable molecular abnormalities, treatment context, response, MRD when informative, and patient preference.

Future models should move beyond static prognostication toward dynamic and treatment-specific prediction, integrating genomic, transcriptomic, epigenomic, single-cell, MRD, and longitudinal clinical information. Such approaches may ultimately identify not only the patients at greatest risk, but also those most likely to benefit from specific therapeutic strategies. 

Returning to the clinical vignette presented at the beginning of this review, the patient with 15% blasts and ASXL1, RUNX1, and SRSF2 mutations illustrates the practical value of this integrated approach. IPSS-M provides the principal estimate of baseline disease risk, while AML-oriented molecular information identifies a myelodysplasia-related genetic context relevant to treatment planning, transplantation assessment, and selected clinical-trial opportunities. Thus, the central question in MDS/AML is no longer whether clinicians should choose between IPSS-M and ELN, but how to combine disease-specific prognostication with treatment-contextual molecular information to guide individualized management. At present, this complementary approach represents the most clinically rational strategy for patients with ICC-defined MDS/AML.

## Figures and Tables

**Table 1:** Distinct Molecular Alterations and Biological Characteristics of ICC-Defined MDS/AML, AML-MR, and De Novo AML.

Molecular Feature	ICC-Defined MDS/AML	AML-MR	De Novo AML	Biological Significance
Dominant disease biology	Transitional overlap state between MDS and AML	AML with a mutation/cytogenetic signature resembling MDS	Primary leukemic disease	Reflects disease ontogeny and evolutionary trajectory
Epigenetic regulators	ASXL1, BCOR, TET2	ASXL1, BCOR, EZH2, TET2	DNMT3A, IDH1/2, TET2	Epigenetic dysregulation contributes to clonal expansion and impaired differentiation
RNA-splicing genes	SRSF2, U2AF1, SF3B1, ZRSR2	SRSF2, U2AF1, ZRSR2	Less frequent	Characteristic of myelodysplastic/secondary-type biology
Transcription factors	RUNX1	RUNX1	Less frequent	Impaired hematopoietic differentiation and leukemic transformation
Cohesin complex genes	STAG2	STAG2, RAD21	Uncommon	Alter chromatin organization and transcriptional regulation
TP53 abnormalities	Enriched	Enriched	Less frequent overall	Associated with genomic instability, complex karyotype, treatment resistance, and adverse outcome
Myelodysplasia-related mutations	Enriched: ASXL1, RUNX1, SF3B1, BCOR, EZH2, STAG2, SRSF2, U2AF1, ZRSR2	Characteristic/defining molecular feature: ASXL1, RUNX1, SF3B1, BCOR, EZH2, STAG2, SRSF2, U2AF1, ZRSR2	Generally uncommon	Reflect secondary-type/myelodysplastic molecular biology
Signaling pathway mutations	Present but not dominant	Variable; may increase with leukemic progression	Common: FLT3, NRAS, KRAS, KIT, PTPN11	Promote proliferation and leukemic expansion
AML-defining genetic abnormalities	Absent by ICC definition	Usually absent within AML-MR categories unless superseded by a higher-priority AML-defining entity	Common; includes NPM1, core-binding factor rearrangements, and other AML-defining abnormalities	Define biologically distinct AML entities
Genomic complexity	Intermediate to high	High	Variable	Reflects clonal evolution and genomic instability
Clonal architecture	Advanced clonal evolution with multiple cooperating lesions	Secondary-type hierarchical clonal evolution	Often dominated by an AML-defining founder/driver clone	Influences disease progression and treatment resistance
Clinical behavior	High-risk overlap disease with features spanning advanced MDS and AML	Adverse-risk AML	Heterogeneous according to molecular subtype	Reflects integration of molecular and clinical characteristics

Comparison of key molecular alterations, genomic features, and biological characteristics across MDS/AML, AML-MR, and de novo AML, highlighting the close relationship between MDS/AML and AML-MR and their distinction from de novo AML.AML-MR may occur in patients without a clinically documented antecedent MDS and is defined by myelodysplasia-related molecular and/or cytogenetic features [9,14,15,17,18,22,23]. Abbreviations: AML, acute myeloid leukemia; AML-MR, acute myeloid leukemia with myelodysplasia-related gene mutations or cytogenetic abnormalities; ICC, International Consensus Classification; MDS/AML, myelodysplastic syndrome/acute myeloid leukemia overlap; MR, myelodysplasia-related.

**Table 2:** Comparative framework of IPSS-M, ELN 2022, proposed modified ELN 2022 for MDS/AML, and ELN 2024 Less-Intensive classification.

Feature	IPSS-M	ELN 2022	Proposed Modified ELN 2022 for MDS/AML	ELN 2024 Less-Intensive
**Primary disease context**	MDS; subsequently validated in ICC-defined MDS/AML [11,19,51]	AML, primarily in the context of intensive therapy [20]	ICC-defined MDS/AML [49]	AML treated with less-intensive therapies [29]
**Purpose**	Molecularly integrated prognostic stratification [19]	Genetic risk stratification and treatment guidance in AML [20]	Disease-specific recalibration of ELN risk stratification for MDS/AML [49]	Treatment-specific genetic risk stratification for less-intensive AML therapy [29]
**Core variables**	Hemoglobin, platelet count, BM blasts, cytogenetics, and mutations in 31 genes [19]	Recurrent AML-associated cytogenetic and molecular abnormalities [20]	Selected cytogenetic and molecular abnormalities recalibrated according to prognostic impact in MDS/AML [49]	Molecular abnormalities associated with outcomes following less-intensive AML therapy [29]
**Risk structure**	Six groups: Very Low, Low, Moderate Low, Moderate High, High, Very High [19]	Three groups: Favorable, Intermediate, Adverse [20]	Three groups: Favorable, Intermediate, Adverse [49]	Three groups: Favorable, Intermediate, Adverse [29]
**Evidence in MDS/AML**	Direct validation demonstrates preserved prognostic discrimination [11,51]	Direct studies show risk-group compression and lower discrimination than IPSS-M [11,51]	Developed specifically for MDS/AML; improves risk distribution and survival discrimination versus ELN 2022 [49]	Recently evaluated in HMA-treated ICC-defined MDS/AML, with significant survival separation [51]
**Strengths in MDS/AML**	Strong disease-specific prognostic discrimination integrating molecular, cytogenetic, blast, and hematologic information [11,19,51]	Established AML-oriented genetic framework relevant when AML-directed strategies are considered [20]	Accounts for disease-specific prognostic effects and reduces ELN 2022 risk compression [49]	Provides treatment-contextual risk information in HMA-treated MDS/AML; numerically higher discrimination than IPSS-M in one retrospective cohort [29,51]
**Limitations in MDS/AML**	Originally developed for MDS rather than specifically for ICC-defined MDS/AML [19]	Developed for AML; adverse-risk clustering and reduced prognostic discrimination in MDS/AML [11,51]	Exploratory model requiring broader external and prospective validation [49]	Evidence remains retrospective, treatment-specific, and based on a relatively small cohort; prospective validation is required [51]
**Clinical role in MDS/AML**	Preferred framework for baseline disease-specific prognostic assessment [11,19,51]	Complementary AML-oriented genetic and treatment-contextual framework [11,20,51]	Promising MDS/AML-specific refinement requiring further validation [49]	Potential treatment-contextual framework for patients receiving HMA-based less-intensive therapy; should not replace IPSS-M for general baseline prognostication [29,51]

Comparative framework of IPSS-M, ELN 2022, proposed modified ELN 2022 for MDS/AML, and ELN 2024 Less-Intensive classification. The table summarizes the disease context, structure, evidence, strengths, limitations, and potential clinical role of each prognostic framework in MDS/AML. Recent evidence suggests that ELN 2024 may provide treatment-contextual prognostic information in HMA-treated MDS/AML, although prospective validation is required. Abbreviations: AML, acute myeloid leukemia; BM, bone marrow; ELN, European LeukemiaNet; HMA, hypomethylating agent; ICC, International Consensus Classification; IPSS-M, Molecular International Prognostic Scoring System; MDS, myelodysplastic syndrome; MDS/AML, myelodysplastic syndrome/acute myeloid leukemia. Note: In the original IPSS-M derivation cohort, 31% of patients with available WHO subtype information were classified as MDS with excess blasts (MDS-EB1/2). However, MDS-EB1 and MDS-EB2 were not reported separately; therefore, the exact proportion of patients with 10–19% bone marrow blasts—and thus the proportion potentially corresponding to the current ICC-defined MDS/AML category—cannot be determined from the published data.

## Data Availability

No new data were created or analyzed in this study. Data sharing is not applicable to this article.
